# Engagement with protective behaviours in the UK during the COVID-19 pandemic: a series of cross-sectional surveys (the COVID-19 rapid survey of adherence to interventions and responses [CORSAIR] study)

**DOI:** 10.1186/s12889-022-12777-x

**Published:** 2022-03-10

**Authors:** Louise E. Smith, Henry W. W. Potts, Richard Amlȏt, Nicola T. Fear, Susan Michie, G. James Rubin

**Affiliations:** 1 Department of Psychological Medicine, King’s College London, Weston Education Centre, Cutcombe Road, London, SE5 9RJ England; 2 NIHR Health Protection Research Unit in Emergency Preparedness and Response, London, England; 3 University College London, Institute of Health Informatics, 222 Euston Road, London, NW1 2DA England; 4 Emergency Response Department Science and Technology, UK Health Security Agency, Behavioural Science Team, Salisbury, Wiltshire, SP4 0JG England; 5 King’s Centre for Military Health Research and the Academic Department of Military Mental Health, King’s College London, London, England; 6 Centre for Behaviour Change, University College London, 1-19 Torrington Place, London, WC1E 7HB England

**Keywords:** Uptake, Face covering, Hand hygiene, Hand cleaning, Social distancing, Physical distancing, Behavioural fatigue

## Abstract

**Background:**

Behaviour is key to suppressing the COVID-19 pandemic. Maintaining behaviour change can be difficult. We investigated engagement with hand cleaning, reducing the number of outings, and wearing a face covering over the course of the pandemic.

**Methods:**

We used a series of 64 cross-sectional surveys between 10 February 2020 and 20 January 2022 (*n* ≈ 2000 per wave). Surveys investigated uptake of hand cleaning behaviours, out of home activity (England only, *n* ≈ 1700 per wave) and wearing a face covering (England only, restricted to those who reported going out shopping in the last week, *n* ≈ 1400 per wave).

**Results:**

Reported hand cleaning has been high throughout the pandemic period (85 to 90% of participants consistently reporting washing their hands thoroughly and regularly with soap and water frequently or very frequently). Out of home activity has mirrored the easing and re-introduction of restrictive measures. Total number of outings were higher in the second national lockdown than in the first and third lockdowns. Wearing a face covering increased steadily between April to August 2020, plateauing until the end of measurement in May 2021, with approximately 80% of those who had been out shopping in the previous week reporting wearing a face covering frequently or very frequently.

**Conclusions:**

Engagement with protective behaviours increased at the start of the pandemic and has remained high since. The greatest variations in behaviour reflected changes to Government rules. Despite the duration of restrictions, people have continued to adopt personal protective behaviours that were intended to prevent the spread of COVID-19.

**Supplementary Information:**

The online version contains supplementary material available at 10.1186/s12889-022-12777-x.

## Background

The COVID-19 pandemic brought with it a new way of life, with a sudden emphasis on behaviours to help prevent the spread of illness, including hand cleaning, reducing contact with others, and wearing a face covering. In the absence of pharmacological interventions, populations around the globe relied on these changes to behaviour to reduce the transmission of COVID-19 [1]. Recommendations to adopt protective behaviours have come at different points during the pandemic. For example, in the UK, the public were urged to adopt respiratory and hand hygiene behaviours by a public information campaign launched on 2 February 2020 [2], to limit their social contact with others on 16 March 2020 [3] and entered the first lockdown on 23 March 2020 [4]. Since this date, a complex series of changes in restrictions has taken place. Rules on wearing a face covering were introduced later. Dates of introduction differed by nation,^1^ but it became mandatory in England on public transport on 15 June 2020 [5], in shops on 24 July [6], and in restaurants on 24 September [7]. Exceptions were made for young children and people with certain medical conditions. All rules on social contact and wearing a face covering were removed on 19 July 2021 [8]. However, the emergence of the Omicron variant of concern brought with it the reimposition of rules on wearing a face covering and working from home [9–11].

Most studies conducted during the pandemic have focused on identifying adherence to certain protective behaviours and identifying factors associated with adherence. Fewer have investigated behaviour over time, likely as these are more time consuming and costly to conduct. One study, conducted between April 2020 and February 2021, indicated that self-reported compliance with Government guidelines decreased in approximately 15% of participants [12]. Market research surveys indicate that levels of wearing a face covering increased between May and September 2020, after which they remained at a steady high rate [13] until the removal of the mandate as part of the 19 July 2021 changes, after which rates of wearing a face covering dropped [14].

Understanding how engagement with protective behaviours varies over time informs actions that may need to be taken to support maintenance of these behaviours long-term. There is also a need to understand whether there is a difference in the rate of adoption between a familiar behaviour such as hand cleaning and a novel (for the UK) behaviour such as wearing a face covering. Previous international research has noted that mandating the wearing of protective equipment (such as bicycle helmets) does not necessarily lead to an immediate widespread uptake [15–17]. Research suggests that behaviours may become habitual in around one to 3 months [18].

In this study, we investigated trends in uptake of protective behaviours during the COVID-19 pandemic in the UK. We used a series of 64 cross-sectional surveys to investigate uptake of three recommended protective behaviours which may influence transmission of COVID-19 over the course of the pandemic. These were: a) hand cleaning (a behaviour recommended before the pandemic that many people are nonetheless poor at performing [19]); b) leaving the home less frequently; and c) wearing a face covering (a novel behaviour for most people in the UK).

## Methods

### Design

Since 28 January 2020, before the first UK cases of COVID-19 were reported, a series of cross-sectional surveys has been conducted by BMG Research and then Savanta on behalf of the Department of Health and Social Care, England. We analysed these data as part of the COVID-19 Rapid Survey of Adherence to Interventions and Responses (CORSAIR) study. Surveys were initially weekly, then usually fortnightly from mid-July 2020. The methods for these surveys have been described in detail elsewhere [20]. In this study, we used data from 10 February 2020 (wave 3) to 20 January 2022 (wave 66).

### Participants

Approximately 2000 participants (people aged 16 years or over living in the UK) completed each survey wave (≈1700 from England per wave). Quota sampling was used, with quotas applied based on age and gender (interlocked) reflecting targets from Office for National Statistics data [21]. From wave 8 of the survey onwards, two specialist research panel providers were used to recruit participants: Respondi (*n* = 50,000) and Savanta (*n* = 31,500). Before then, participants were recruited solely from the Respondi panel. Participants could complete multiple survey waves, but after having completed the survey once were excluded from the subsequent three waves. An error with one of the survey panels meant this exclusion rule was not applied in the first waves of data collection, leading to a small number of people (at least *n* = 133, 0.2% of sample) completing the survey nine times or more up to 18 November 2020 (wave 30). We have adjusted statistically for participants appearing in multiple waves, where possible. Due to an error, some repeat participants were not identified in the data available. We were unable to identify repeat participants from one of the panels before 20 September 2021 (wave 58). The impact of adjusting for repeat participants on the results is minimal. Therefore, this inability to identify some repeat participants should not have any material impact on results. Participants were reimbursed for having completed the survey in points, which could be redeemed as cash, gift vouchers or charitable donations (up to 70p per survey).

### Measures

Questionnaire items reported were developed for the CORSAIR study. Full survey items for outcome measures are reported in the supplementary materials.

Hand cleaning: From 10 January 2020 (wave 3), participants were asked if they had washed their hands thoroughly and regularly with soap and water in the past 7 days. Response options were “done this, same amount as usual,” “done this, more than usual,” “not done this,” and “not applicable.” Between 27 April 2020 (wave 14) to 13 May 2020 (wave 16), the sample was split, with half the sample being asked to respond using the original options, and half the sample being asked to respond on a five-point scale from “never” to “very frequently.” From 18 May 2020 (wave 17) onwards, all participants responded on the five-point scale. From 26 October 2020 (wave 31) onwards, the item was amended so that participants were asked if they had “washed [their] hands thoroughly and regularly with soap and water, or used hand sanitising gel”. We created binary variables to indicate if people reported washing their hands frequently or very frequently (compared to never, rarely or occasionally). This item was removed from the survey after 19 May 2021 (wave 50). For these variables, “not applicable” was coded as missing (*n* = 847, 0.9%).

Number of outings: Participants were asked to state the number of times they had been out of their home in the last 7 days to go: to the shops, for groceries/pharmacy; to the shops, for things other than groceries/pharmacy; for a walk or some other exercise; to spend time outdoors for recreational purposes; out to work; to meet up with friends and/or family they did not live with; and to a restaurant, café or pub. These questions were introduced to the surveys on 30 March 2020 (wave 10), with the exception of spending time outdoors for recreational purposes, which was introduced on 18 May 2020 (wave 17), and going out to a restaurant, café or pub, which was introduced on 6 July 2020 (wave 24). This measure was amended on 1 June 2021 (wave 51), and from this date on, asked participants to state the number of times they had done each of the following activities in the past 7 days, including having: been to the shops, for groceries/pharmacy; been to the shops, for things other than groceries/pharmacy; spent time outdoors for exercise or recreational purposes (including to sit in parks etc.); left the house to go out to work (number of days); met up with friends and/or family that they did not live with; and been to a restaurant, café or pub. To investigate total number of outings where people were likely to come into close contact with someone from another household indoors, we created three separate variables. The first variable summed outings for shopping and to see friends or family from another household to give a “total number of outings” variable. As the item investigating outings to hospitality venues was introduced later, we created a second variable that also included going out to a restaurant, café or pub. A third variable, summing outings for shopping, to see friends or family from another household, visiting hospitality venues and for work was also created, but only calculated for those who reported being in employment.

Wearing a face covering: Participants were asked if they had worn a professional face mask or a homemade, cloth or improvised face covering (such as a scarf) when out and about in the last 7 days on a five-point scale from “never” to “very frequently.” Between 27 April 2020 (wave 14) to 13 May 2020 (wave 16), this question was only asked to half the sample. From 18 May 2020 (wave 17), all participants were asked to respond on the five-point scale. We coded participants as wearing a face covering if they selected “very frequently” or “frequently” for either or both of the face covering items. On 26 October 2020 (wave 31), the two items were replaced by a single item asking how frequently participants had “worn a face mask or another face covering (such as a scarf) when out and about”. This item was removed from the survey after 19 May 2021 (wave 50). For these items, we treated answers of “not applicable” as not having worn a face covering frequently or very frequently (*n* = 432, 0.8%).

#### Participant characteristics

Participants were asked to report their sex, age, ethnicity, highest level of educational or professional attainment, employment status and socio-economic grade [22]. Region was derived from participants’ postcodes.

### Ethics

This work was conducted as part of a service evaluation of the marketing and communications run by the Department of Health and Social Care, and so did not require ethical approval. We sought advice from the Psychiatry, Nursing and Midwifery Research Ethics Office, King’s College London and they confirmed this position.

### Analysis

For analyses investigating hand cleaning, we used data from all participants completing the survey between 10 February 2020 and 19 May 2021 (waves 3 to 50), as these were the dates that the relevant outcome question was included (*n* = 95,998 responses).

Due to differences in rules on outings in the devolved nations, we restricted the sample to include only participants living in England (included in surveys between 30 March 2020 and 20 January 2022 [waves 10 to 66], *n* = 100,736 responses) for analyses of outings. For the number of outings for work, we restricted analyses to those who reported they were in full-time, part-time or self-employment (*n* = 54,504 responses).

For analyses of wearing a face covering, due to differences in rules in the devolved nations, we restricted the sample only to those in England who reported having been out shopping in the last 7 days (included in survey between 27 April 2020 and 19 May 2021 [waves 14 to 50], *n* = 52,285 responses).

We described engagement with protective behaviours descriptively and graphically, using line graphs to show the percentage of people who reported engaging in hand cleaning frequently or very frequently, the number of times people reported going out in the last week for various reasons, and the percentage of people who reported wearing a face covering frequently or very frequently. For each data point, we calculated 95% confidence intervals.

For analyses investigating hand washing and wearing a face covering, phrasing of the question changed on 26 October 2020 (wave 31). We compared data from 28 September to 11 November 2020 (waves 29 to 32; last two waves of the original compared to first two waves of the updated question wording; no repeat respondents) using a χ^2^-test, to investigate whether responses for the different question wordings were comparable.

We used generalised estimating equations (GEEs) to investigate whether engagement with protective behaviours differed by survey wave, accounting for repeat respondents where possible. For binary outcomes (hand washing, wearing a face covering), we used logistic regression analyses, reporting odds ratios (ORs). For count data (total outings), we used negative binomial regressions, reporting incidence rate ratios (IRRs). We also investigated whether total outings differed between lockdown periods. As participants reported on their behaviour in the previous 7 days, we selected waves where the reporting period was solely contained under lockdown restrictions (first lockdown: 30 March to 6 May 2020, waves 10 to 15; second lockdown: 16 November to 2 December 2020, waves 33 to 35; third lockdown: 25 January to 24 February 2021, waves 42 to 44).

## Results

### Respondent characteristics

Respondent characteristics are reported in Table 1. Approximately half of responses were from women (53%), with a mean age of 48.5 years. Respondents’ ethnicity was broadly reflective of the general population [23].

**Table 1:** Respondent characteristics

Respondent characteristics	Level	N	Percentage (%)
Sex	Male	61,470	46.4
	Female	70,574	53.3
	Prefer not to self-describe	310	0.2
	Prefer not to say	106	0.1
Age (years)	Range = 16 to over 100	Mean = 48.5, standard deviation = 17.8	
Nation	England	112,882	85.2
	Scotland	10,457	7.9
	Wales	6672	5.0
	Northern Ireland	2449	1.8
Ethnicity	White British	111,275	84.0
	White other	8241	6.2
	Mixed	2929	2.2
	Asian / Asian British	6114	4.6
	Black / Black British	2610	2.0
	Arab / other	539	0.4
	Prefer not to say	752	0.6
Employment status	Not working	59,631	45.0
	Working	71,191	53.7
	Prefer not to say	1638	1.2
Socio-economic grade	ABC1	72,538	54.8
	C2DE	57,107	43.1
	Not categorised (highest earning profession not specified)	2815	2.1
Highest educational or professional qualification	GCSE/vocational/A-level/No formal qualifications	87,814	66.3
	Degree or higher (Bachelors, Masters, PhD)	44,646	33.7

### Hand cleaning

**Fig. 1:**
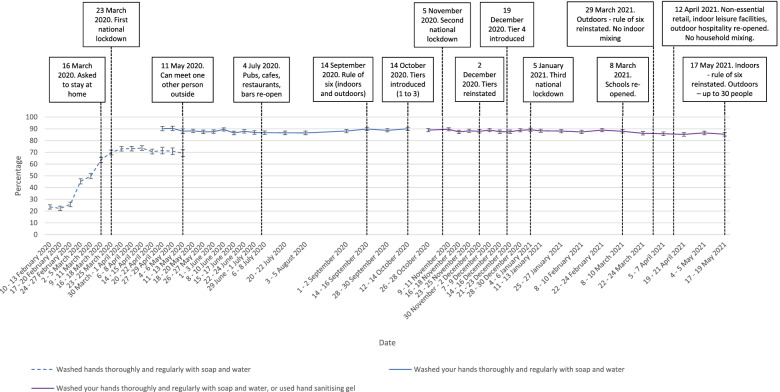
Graph depicting the percentage of people who reported washing their hands thoroughly and regularly, between February 2020 and May 2021. Dashed lines indicate those who reported hand cleaning more than usual; solid lines indicate those who reported hand cleaning frequently or very frequently. Error bars are 95 confidence intervals

The percentage of people who reported washing their hands thoroughly and regularly more than usual increased sharply between February and April 2020 from 22% to approximately 70% (Fig. 1; χ^2^(12) = 2725.8, *p* < .001; supplementary materials). The percentage of people reporting washing their hands thoroughly and regularly frequently or very frequently remained relatively stable at 90% from May 2020, with small decreases seen in April and May 2021. Although this change was statistically significant (χ^2^(36) = 117.2, *p* < .001; supplementary materials), in practice, there was only a small change in the percentage of people engaging in thorough hand washing (approximately 90 to 85%).

Changing the question wording made no difference to results (χ^2^(3) = 2.5, *p* = 0.48).

### Number of outings

**Fig. 2:**
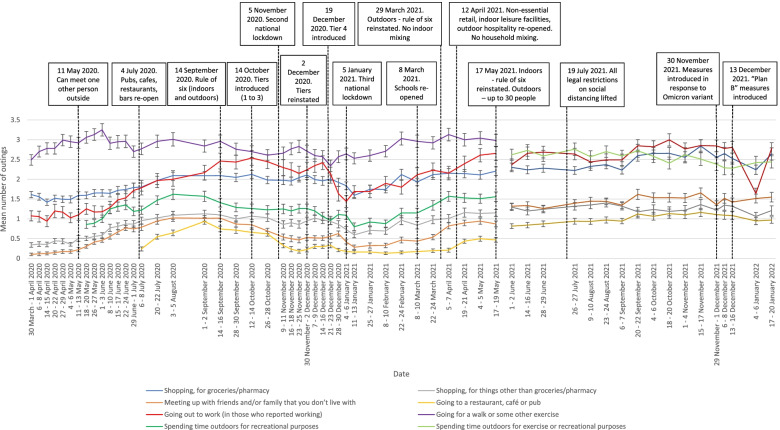
Graph depicting the mean number of times that people reported going out for different reasons in the last seven days, between March 2020 and January 2022. Error bars are 95% confidence intervals

During the initial period of restrictions, outings to meet up with people from another household or go to shops for non-essential goods (both prohibited activities) were low (see Fig. 2). These increased, roughly in line with visits to restaurants, cafes and pubs when the item was introduced, until September 2020, when new restrictions on the maximum number of people able to meet and a new work from home recommendation were issued. During the second (November 2020) and third (January 2021 to March 2021) national lockdowns, these rates fell but remained higher than during the first lockdown. Leaving home to go to the shops for groceries or pharmacy goods has shown a similar general pattern, albeit at higher overall levels.

Leaving home to go to work was initially at very low levels during the first lockdown, despite being allowed where necessary. This showed an increase over time from May 2020 until September 2020, when a request to work from home where possible was once again introduced. There was no change between September to October 2020, after which point it decreased during the second lockdown (November 2020). Levels of going out to work increased over the third lockdown (January 2021 to June 2021), then stabilising at the highest rate in the pandemic in September to December 2021. Levels of going out to work over the festive period fell sharply, but returned back to similar levels in January 2022.

The most common reason for leaving home throughout the pandemic was to go for a walk or some other exercise. This showed an initial increase from March to June 2020, then a slight decrease in early June and again since late September 2020. Levels of spending time outdoors for recreational purposes was lower, but showed increases during times with fewer restrictions. From June 2021, number of times spent outdoors for exercise or recreational purposes has consistently been among the most common reasons for leaving home.

**Fig. 3:**
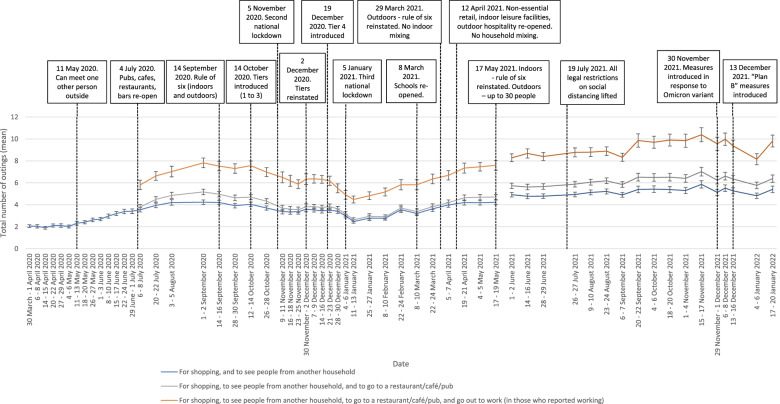
Graph depicting the total number of outings in the last seven days, between March 2020 and January 2022. Error bars are 95% confidence intervals

Total number of outings increased steadily from March to September 2020, after which it fell (see Fig. 3). Total outings during the second (November 2020) and third (January to March 2021) national lockdowns were higher than during the first lockdown in (March to May 2020; χ^2^(2) = 574.4, *p* < .001; supplementary materials). Outings during the third lockdown were lower than those seen in the second lockdown (IRRs 0.86–0.87 (95% CIs 0.81–0.82 to 0.91–0.92), *ps* < .001; supplementary materials). After that, rates steadily climbed, until January 2021 after “Plan B” measures were introduced [10].

### Wearing a face covering

**Fig. 4:**
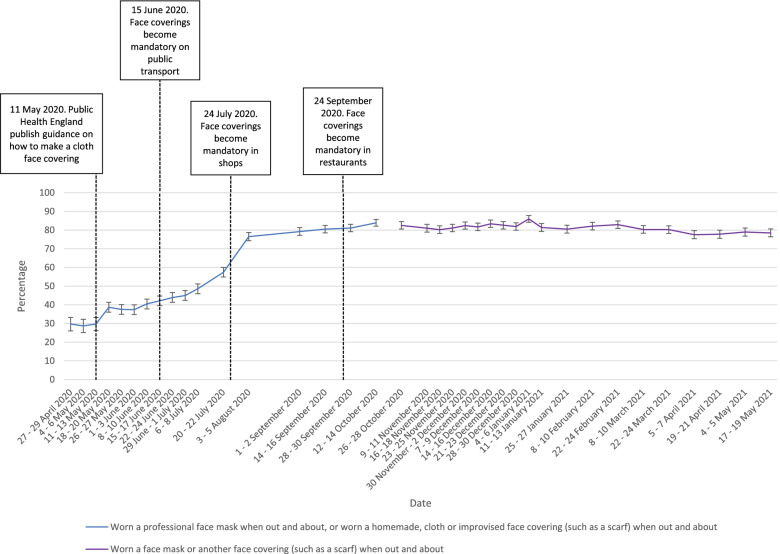
Graph depicting the percentage of people who reported wearing a face covering frequently or very frequently when out and about in the last seven days in those who reported having been shopping, between April 2020 and May 2021. Error bars are 95% confidence intervals

The percentage of people reporting wearing a face covering frequently or very frequently differed significantly between April 2020 and May 2021 (χ^2^(36) = 8118.2, *p* < 0.001; supplementary materials). Rates of wearing a face covering were stable from late April to mid-May 2020, when guidance on face coverings was produced (see Fig. 4; ORs 0.98–1.07 (95% CIs 0.79–0.87 to 1.22–1.32, *ps* = 0.52–0.88; supplementary materials). This was followed by a sudden, small increase. Following a plateau, discussion and then a series of laws making coverings mandatory in different locations there was a steady increase between early June and August. Wearing of face coverings stabilised at around 80% between September 2020 to March 2021, after which they decreased slightly (χ^2^(19) = 82.3, *p* < .001). Despite a statistically significant difference, in practice rates only fell by approximately 4% (approximately 82 to 78%).

Changing the question wording made no difference to results (χ^2^(3) = 5.8, *p* = 0.12).

## Discussion

Key recommended behaviours increased at the start of the COVID-19 pandemic and have been maintained at high levels as the pandemic has progressed. Self-reported hand cleaning showed a marked increase in the first few weeks of the pandemic and remained at high levels thereafter. Despite a statistically significant decrease in rates of frequent handwashing between April and May 2021, rates of frequent hand washing only changed by a small amount (approximately 85% frequent hand washing compared to 90%). In a separate survey, rates of people “improving personal hygiene” also increased steeply between March and May 2020, then fell steadily until January 2022 [13]. This difference in results may be explained in part by the phrasing of the survey item, with people no longer “improving” their personal hygiene measures, but maintaining them at a high level, as suggested by our results. Due to the use of self-report measures, it is likely that rates are over-estimates of engagement with protective behaviours.

The number of outings reported reflects a more complex picture but broadly mirrors UK Government guidance, rising as restrictions were eased over the summer of 2020, falling as they were re-introduced in September (rule-of-six), October (tiers), and November (second lockdown) 2020, decreasing further with the start of the third national lockdown in January 2021, and then rising steadily with the removal of all restrictions on social mixing in summer 2021. Objective measures corroborate these findings, with evidence from mobile phone location data indicating that people’s out of home activities have changed in line with national restrictions [24–26]. Other data from our group also suggest that the biggest influence in patterns of social mixing is guidance in place at the time [27]. The one notable exception is leaving home to go to work. In both the UK’s first lockdown (March to May 2020) and England’s second lockdown (November 2020), people were asked to work from home, but to go to work if that was not possible [28, 29]. However, the impact on work from home was weaker in November and self-reported rates of going out to work were higher in November than in March. We also saw a sharp drop in the percentage of people reporting having been out to work at the start of January 2022. Rather than reflecting changes in guidance (“Plan B”), this is likely due to the closure of many workplaces over the festive period, as demonstrated by returns to higher levels later on in January 2022. Our findings present an insight into complex behaviour changes in routine out of home activities over the course of the pandemic.

The total number of outings during the second (November 2020) and third (January to February 2021) English lockdowns were higher than during the first lockdown (March to May 2020). The second lockdown was less strict than the first lockdown, with outings to meet an individual socially outside being allowed, and schools, universities and more workplaces remaining open [28, 29].

For wearing a face covering, a behaviour that was not commonplace in the UK before the pandemic, uptake increased slowly at the start of the pandemic until September 2020 and subsequently remained at a stable high level, followed by a slight decrease in April to May 2021. This is in line with other data collected between March 2020 and January 2021 [30]. Data collected after the period of data collection for this study show that the percentage of people wearing a face covering in shops decreased after the mandate was lifted, and then increased after the mandate was reintroduced in response to the emergence of the Omicron variant of concern [11, 13, 14].

Rates of hand cleaning increased sharply at the beginning of the pandemic, while rates of wearing face coverings increased slowly. This could have been due to lack of social norms and low perceived effectiveness of wearing face coverings. These factors are theoretically associated with uptake of health behaviours (e.g. as part of reflective motivation in the COM-B model [31]). At the start of the pandemic, reflective motivation was strongly associated with adopting good hygiene practices [32]. Greater perceived effectiveness and social norms have been shown to be associated with uptake of protective behaviours during the COVID-19 pandemic [33], including wearing a face covering [34–36].

The term “behavioural fatigue” has come to be used colloquially as a slightly amorphous ‘catch-all’ term for an inevitable decline in adherence to protective behaviours [37]. Despite its common use in the media and implication in previous policy decisions (e.g. [38]), there is little evidence to support this notion. In our data, uptake of behaviours tended to increase and was then maintained over a long period of time. Other studies have also found this pattern of behaviour [13, 30, 36, 39]. Where studies have found decreases in engagement with protective behaviours, this has only been in a small percentage of people [12].

The causes of changes in engagement with protective behaviours over time are unclear [40]. The COM-B model suggests that decreases in engagement with protective behaviours are the result of decreasing capability, opportunity and/or motivation [31]. While motivation to engage with certain protective behaviours may have decreased, capability and opportunity have likely grown over the course of the pandemic. For example, we now know more about COVID-19 transmission than at the start of the pandemic (psychological capability). Physical opportunity may also have increased, with many indoor shared spaces making hand sanitiser freely available and configuring their settings to allow physical distancing. Wearing a face covering, previously not a common behaviour in the UK, has now become commonplace, increasing social norms (social opportunity). Over the course of the pandemic, there has been ample time for protective behaviours to become routine or even habitual, a process which takes weeks or months [18].

Strengths of this study include the use of a large sample to investigate trends in engagement with protective behaviours during the pandemic. The measurement of multiple out of home activities gives insight into how complex routine behaviours have changed. This study has some limitations. Participants were recruited into the study using quota sampling to ensure that the sample was broadly representative of the UK population, based on Office for National Statistics data [21]. Respondents were slightly more likely to be women than in the general population. We cannot be sure that the views of survey respondents are representative of the general population [41, 42]. Data were self-reported and may be influenced by social desirability and recall biases. The phrasing of some of the questions may encourage positive reporting. In another study, people were less likely to report washing their hands if the question was framed negatively compared to positively [43]. The phrasing of some items changed over the course of the surveys, making direct comparisons difficult. Some items introduced a degree of subjectivity (e.g. washing hands “thoroughly and regularly”). Survey questions may be unable to detect subtle changes in behaviour, for example, the duration of hand washing, that may still have implications for viral transmission.

## Conclusions

While self-reported rates of hand cleaning, out of home activity and wearing a face covering should be taken with caution, trends in the data paint a useful picture of engagement with protective behaviours among the UK population over a long period of time. Our results indicate that hand cleaning increased rapidly at the start of the pandemic and remained high since. Adoption of a previously uncommon behaviour in the UK, wearing a face covering, increased more slowly, but has remained at a stable high rate while a mandate was in operation. Out of home activity reflected UK Government guidance in place at the time. Results have important implications for policy makers, indicating that engagement with protective behaviours can be maintained over a long period of time, and that behaviour largely follows rules in place.

## Supplementary Information

**Additional file 1.**

## Data Availability

The data that support the findings of this study are available from the Department of Health and Social Care but restrictions apply to the availability of these data, which were used under license for the current study, and so are not publicly available. Data are however available from the authors upon reasonable request and with permission of the Department of Health and Social Care.

## Abbreviations

UK: United Kingdom

## Footnotes

1 Mandatory use of face coverings differed by nation. England: 15 June 2020 – public transport; 24 July 2020 – shops; 24 September 2020 – restaurant workers and in restaurants. Scotland: 22 June 2020 – public transport; 10 July 2020 – shops; 14 August 2020 – indoor public spaces including restaurants. Wales: 27 July 2020 – public transport; 14 September 2020 – indoor public spaces including shops and restaurants. Northern Ireland: 10 July 2020 – public transport; 10 August 2020 – shops; 14 October 2020 – restaurants.
