# Correction: Engagement with protective behaviours in the UK during the COVID-19 pandemic: a series of cross-sectional surveys (the COVID-19 rapid survey of adherence to interventions and responses [CORSAIR] study)

**DOI:** 10.1186/s12889-022-13472-7

**Published:** 2022-06-10

**Authors:** Louise E. Smith, Henry W. W. Potts, Richard Amlȏt, Nicola T. Fear, Susan Michie, G. James Rubin

**Affiliations:** 1 Department of Psychological Medicine, King’s College London, Weston Education Centre, Cutcombe Road, London, SE5 9RJ England; 2 NIHR Health Protection Research Unit in Emergency Preparedness and Response, London, England; 3 University College London, Institute of Health Informatics, 222 Euston Road, London, NW1 2DA England; 4 Emergency Response Department Science and Technology, UK Health Security Agency, Behavioural Science Team, Salisbury, Wiltshire SP4 0JG England; 5 King’s Centre for Military Health Research and the Academic Department of Military Mental Health, King’s College London, London, England; 6 Centre for Behaviour Change, University College London, 1-19 Torrington Place, London, WC1E 7HB England

**Correction: BMC Public Health 22, 475 (2022)**

**https://doi.org/10.1186/s12889-022-12777-x**

The original publication of this article [1] contained some errors as a result of the dataset provided to the authors.

There was a problem concerning the variable denoting responder ID. For one panel used, all respondents were assigned unique IDs each time they completed a survey, regardless of whether they had previously completed the survey.

Therefore, repeat respondents were not identified. This affected data in waves 8 to 57 (inclusive). The authors have since worked with the market research companies to rectify this problem. For another article, re-running generalised estimating equations (GEEs) using corrected responder IDs did not meaningfully change results [2] .

This should not affect the results nor conclusions of this article [1].

**Funding statement**

This work was funded by the National Institute for Health Research (NIHR) Health Services and Delivery Research programme (NIHR project reference number (11/46/21)). Surveys were commissioned and funded by Department of Health and Social Care (DHSC), with the authors providing advice on the question design and selection. LS, RA and GJR are supported by the National Institute for Health Research Health Protection Research Unit (NIHR HPRU) in Emergency Preparedness and Response, a partnership between the UK Health Security Agency, King’s College London and the University of East Anglia. RA is also supported by the NIHR HPRU in Behavioural Science and Evaluation, a partnership between the UK Health Security Agency and the University of Bristol. HWWP has received funding from Public Health England and NHS England. NTF is part funded by a grant from the UK Ministry of Defence. The views expressed are those of the authors and not necessarily those of the NIHR, UK Health Security Agency, the Department of Health and Social Care or the Ministry of Defence. The Department of Health and Social Care funded data collection (no grant number).

**Competing interests statement**

All authors had financial support from NIHR for the submitted work. RA is an employee of the UK Health Security Agency; HWWP received additional salary support from Public Health England and NHS England; HWWP receives consultancy fees to his employer from Ipsos MORI and has a PhD student who works at and has fees paid by Astra Zeneca. At the time of writing GJR is acting as an expert witness in an unrelated case involving Bayer PLC, supported by LS. NTF is a participant of an independent group advising NHS Digital on the release of patient data. All authors were participants of the UK’s Scientific Advisory Group for Emergencies or its subgroups.
