# Supplementary material for: Engagement with protective behaviours in the UK during the COVID-19 pandemic: a series of cross-sectional surveys (the COVID-19 rapid survey of adherence to interventions and responses [CORSAIR] study)
Source: BMC Public Health. 2022 Mar 10;22:475. doi: 10.1186/s12889-022-12777-x (PMC8907902; doi:10.1186/s12889-022-12777-x)
Supplement: Supplementary file 1 — Additional file 1. [file 12889_2022_12777_MOESM1_ESM.docx]

# Supplementary materials 1. Survey items.

Hand cleansing and wearing a face covering

*Asked in wave 3 to wave 16. From wave 3 to wave 13 all participants answered this question. The sample was split in wave 14 to wave 16 with half of participants answering this question.*

**In the past seven days have you…**

- Washed your hands thoroughly and regularly with soap and water
- Done this, same amount as usual
- Done this, more than usual
- Not done this
- Not applicable

*Asked in wave 14 to wave 30. The sample was split in wave 14 to wave 16 with half of participants answering this question. From wave 17 to wave 30 all participants answered this question.*

**In the past seven days have you …**

- Washed your hands thoroughly and regularly with soap and water
- Worn a professional face mask when out and about
- Worn a homemade, cloth or improvised face mask (such as a scarf) when out and about
- Very Frequently
- Frequently
- Occasionally
- Rarely
- Never
- Not applicable

*Asked in wave 31 to wave 50 to all participants.*

**In the past seven days have you …**

- Washed your hands thoroughly and regularly with soap and water, or used hand sanitising gel
- Worn a face mask or another face covering (such as a scarf) when out and about
- Very Frequently
- Frequently
- Occasionally
- Rarely
- Never
- Not applicable

Number of outings

*Asked from wave 10 to wave 50 to all participants.*

**Please enter the number of times you have been out of your home in the last seven days, for each of the following reasons?**

**If you have not left your home for this reason, please write 0**

RANDOMISE ORDER

- To go to the shops, for groceries/pharmacy
- To go to the shops, for things other than groceries/pharmacy
- To go for a walk or some other exercise
- To spend time outdoors for recreational purposes (including to sit in parks etc.) *[Item added in wave 17]*
- To go out to work
- To meet up with friends and/or family that you don’t live with
- To go to a restaurant, café or pub *[Item added in wave 24]*

OPEN - NUMERICAL VALUE ONLY

*Asked from wave 51 onwards to all participants.*

**Q8_C. How many times have you done each of the following activities in the past seven days?**

**Please give an approximate number if you are unsure. If you have not done the activity at all in the last seven days, please write 0.**

RANDOMISE ORDER

- Been to the shops, for groceries/pharmacy
- Been to the shops, for things other than groceries/pharmacy
- Spent time outdoors for exercise or recreational purposes (including to sit in parks etc.)
- Left the house to go out to work (number of days)
- Met up with friends and/or family that you don’t live with
- Been to a restaurant, café or pub

OPEN - NUMERICAL VALUE ONLY

# Supplementary materials 2. Associations with survey wave.

Results reported here use are for all participants with a valid individual response ID. Individual response IDs were only collected from 17 February 2020 (wave 4) onwards, therefore all participants from data collected 10 to 13 February 2020 (wave 3, n=2006) were excluded. Individual response ID was not collected for a small number of participants in subsequent waves, participants were excluded (n=743, 0.6%, wave 4 to 66). Therefore, point percentages may differ slightly from those reported in the main text.

All generalised estimating equations (GEEs) were run using an unstructured correlation matrix, unless otherwise specified.

Table 1. Associations between more frequent hand washing and survey wave, between February and May 2020.

| Survey wave | Not more frequent hand washing [total n=9,442], n (%) | More frequent hand washing [total n=13,315], n (%) | Odds ratio (95% CI) | p-value |
| --- | --- | --- | --- | --- |
| Overall | - | - | χ^2^(12)=2725.8 | <0.001 |
| 17 to 20 February 2020 (wave 4)† | 1530 (77.7) | 440 (22.3) | Reference | Reference |
| 24 to 27 February 2020 (wave 5) | 1468 (74.3) | 508 (25.7) | 1.20 (1.04 to 1.39) | 0.01 |
| 2 to 5 March 2020 (wave 6) | 1085 (54.6) | 901 (45.4) | 2.88 (2.51 to 3.30) | <0.001 |
| 9 to 11 March 2020 (wave 7) | 997 (50.3) | 987 (49.7) | 3.42 (2.98 to 3.92) | <0.001 |
| 16 to 18 March 2020 (wave 8) | 723 (36.5) | 1259 (63.5) | 6.04 (5.26 to 6.94) | <0.001 |
| 23 to 25 March 2020 (wave 9) | 600 (30.1) | 1392 (69.9) | 8.04 (6.98 to 9.26) | <0.001 |
| 30 March to 1 April 2020 (wave 10) | 536 (27.1) | 1444 (72.9) | 9.33 (8.08 to 10.78) | <0.001 |
| 6 to 8 April 2020 (wave 11) | 528 (26.8) | 1440 (73.2) | 9.46 (8.18 to 10.93) | <0.001 |
| 14 to 15 April 2020 (wave 12) | 521 (26.4) | 1455 (73.6) | 9.60 (8.31 to 11.08) | <0.001 |
| 20 to 22 April 2020 (wave 13) | 580 (29.4) | 1393 (70.6) | 8.36 (7.25 to 9.64) | <0.001 |
| 27 to 29 April 2020 (wave 14) | 281 (28.5) | 705 (71.5) | 8.63 (7.26 to 10.24) | <0.001 |
| 4 to 6 May 2020 (wave 15) | 293 (29.2) | 712 (70.8) | 8.36 (7.06 to 9.90) | <0.001 |
| 11 to 13 May 2020 (wave 16) | 300 (30.6) | 679 (69.4) | 8.04 (6.79 to 9.53) | <0.001 |

†Hand washing behaviour was measured from 10 February 2020 (wave 3) onwards. Individual response IDs were collected from 17 February 2020 (wave 4) onwards. As generalised estimating equations (GEEs) require individual response IDs, we are unable to include wave 3 data in GEE analyses.

Table 2. Associations between frequent hand washing and survey wave, between April 2020 and May 2021.

| Survey wave | Infrequent hand washing [total n=8,637], n (%) | Frequent hand washing [n=62,514], n (%) | Odds ratio (95% CI) | p-value |
| --- | --- | --- | --- | --- |
| Overall | - | - | χ^2^(36)=117.2 | <0.001 |
| 27 to 29 April 2020 (wave 14) | 97 (9.8) | 895 (90.2) | Reference | Reference |
| 4 to 6 May 2020 (wave 15) | 96 (9.6) | 900 (90.4) | 1.11 (0.84 to 1.47) | 0.47 |
| 11 to 13 May 2020 (wave 16) | 120 (12.0) | 877 (88.0) | 0.89 (0.68 to 1.17) | 0.40 |
| 18 to 20 May 2020 (wave 17) | 232 (11.7) | 1749 (88.3) | 0.92 (0.72 to 1.17) | 0.48 |
| 26 to 27 May 2020 (wave 18) | 249 (12.6) | 1730 (87.4) | 0.84 (0.66 to 1.07) | 0.16 |
| 1 to 3 June 2020 (wave 19) | 244 (12.3) | 1732 (87.7) | 0.88 (0.69 to 1.11) | 0.27 |
| 8 to 10 June 2020 (wave 20) | 208 (10.5) | 1772 (89.5) | 1.02 (0.80 to 1.29) | 0.90 |
| 15 to 17 June 2020 (wave 21) | 267 (13.5) | 1713 (86.5) | 0.78 (0.61 to 0.98) | 0.03 |
| 22 to 24 June 2020 (wave 22) | 240 (12.1) | 1744 (87.9) | 0.87 (0.69 to 1.11) | 0.26 |
| 29 June to 1 July 2020 (wave 23) | 260 (13.1) | 1721 (86.9) | 0.80 (0.63 to 1.02) | 0.07 |
| 6 to 8 July 2020 (wave 24) | 264 (13.3) | 1721 (86.7) | 0.81 (0.64 to 1.03) | 0.08 |
| 20 to 22 July 2020 (wave 25) | 265 (13.3) | 1722 (86.7) | 0.78 (0.61 to 0.98) | 0.03 |
| 3 to 5 August 2020 (wave 26) | 265 (13.4) | 1714 (86.6) | 0.77 (0.61 to 0.97) | 0.03 |
| 1 to 2 September 2020 (wave 27) | 238 (11.9) | 1760 (88.1) | 0.88 (0.69 to 1.13) | 0.31 |
| 14 to 16 September 2020 (wave 28) | 203 (10.2) | 1794 (89.8) | 1.04 (0.82 to 1.33) | 0.74 |
| 28 to 30 September 2020 (wave 29) | 228 (11.3) | 1796 (88.7) | 0.93 (0.73 to 1.19) | 0.59 |
| 12 to 14 October 2020 (wave 30) | 205 (10.0) | 1854 (90.0) | 1.08 (0.84 to 1.38) | 0.55 |
| 26 to 28 October 2020 (wave 31) | 226 (11.1) | 1805 (88.9) | 0.94 (0.74 to 1.20) | 0.63 |
| 9 to 11 November 2020 (wave 32) | 210 (10.4) | 1811 (89.6) | 1.02 (0.80 to 1.31) | 0.85 |
| 16 to 18 November 2020 (wave 33) | 258 (12.6) | 1784 (87.4) | 0.83 (0.66 to 1.06) | 0.13 |
| 23 to 25 November 2020 (wave 34) | 236 (11.7) | 1775 (88.3) | 0.90 (0.71 to 1.14) | 0.38 |
| 30 November to 2 December 2020 (wave 35) | 246 (12.1) | 1789 (87.9) | 0.88 (0.69 to 1.11) | 0.28 |
| 7 to 9 December 2020 (wave 36) | 232 (11.1) | 1853 (88.9) | 0.95 (0.75 to 1.21) | 0.68 |
| 14 to 16 December 2020 (wave 37) | 249 (12.4) | 1757 (87.6) | 0.84 (0.66 to 1.07) | 0.16 |
| 21 to 23 December 2020 (wave 38) | 248 (12.5) | 1742 (87.5) | 0.84 (0.66 to 1.06) | 0.15 |
| 28 to 30 December 2020 (wave 39) | 226 (11.2) | 1786 (88.8) | 0.94 (0.74 to 1.20) | 0.62 |
| 4 to 6 January 2021 (wave 40) | 219 (10.8) | 1808 (89.2) | 0.99 (0.77 to 1.26) | 0.93 |
| 11 to 13 January 2021 (wave 41) | 232 (11.7) | 1757 (88.3) | 0.91 (0.71 to 1.15) | 0.43 |
| 25 to 27 January 2021 (wave 42) | 237 (11.9) | 1759 (88.1) | 0.89 (0.70 to 1.13) | 0.35 |
| 8 to 10 February 2021 (wave 43) | 254 (12.7) | 1752 (87.3) | 0.82 (0.65 to 1.05) | 0.11 |
| 22 to 24 February 2021 (wave 44) | 223 (11.1) | 1779 (88.9) | 0.96 (0.75 to 1.22) | 0.71 |
| 8 to 10 March 2021 (wave 45) | 242 (12.1) | 1757 (87.9) | 0.86 (0.68 to 1.09) | 0.22 |
| 22 to 24 March 2021 (wave 46) | 281 (13.8) | 1762 (86.2) | 0.75 (0.59 to 0.95) | 0.02 |
| 5 to 7 April 2021 (wave 47) | 284 (14.2) | 1712 (85.8) | 0.73 (0.57 to 0.92) | 0.01 |
| 19 to 21 April 2021 (wave 48) | 293 (14.7) | 1695 (85.3) | 0.69 (0.55 to 0.88) | 0.002 |
| 4 to 5 May 2021 (wave 49) | 268 (13.5) | 1724 (86.5) | 0.77 (0.61 to 0.98) | 0.03 |
| 17 to 19 May 2021 (wave 50) | 292 (14.6) | 1713 (85.4) | 0.69 (0.55 to 0.88) | 0.002 |

Table 3. Associations between total outings for shopping, and to see people from another household and survey wave, between April 2020 and May 2021.

| Survey wave | Number of responses, mean number of outings, SD [total n=70,493] | Incidence rate ratio (95% CI) | p-value |
| --- | --- | --- | --- |
| Overall | - | χ^2^(40)=2559.7 | <0.001 |
| 30 March to 1 April 2020 (wave 10) | N=1739, M=2.1, SD=2.7 | Reference | Reference |
| 6 to 8 April 2020 (wave 11) | N=1738, M=2.0, SD=3.3 | 0.95 (0.86 to 1.04) | 0.24 |
| 14 to 15 April 2020 (wave 12) | N=1742, M=1.9, SD=2.8 | 0.90 (0.82 to 0.98) | 0.01 |
| 20 to 22 April 2020 (wave 13) | N=1735, M=2.1, SD=3.1 | 0.98 (0.90 to 1.07) | 0.71 |
| 27 to 29 April (wave 14) | N=1698, M=2.1, SD=3.3 | 0.98 (0.90 to 1.08) | 0.71 |
| 4 to 6 May 2020 (wave 15) | N=1724, M=2.0, SD=2.8 | 0.95 (0.87 to 1.03) | 0.24 |
| 11 to 13 May 2020 (wave 16) | N=1742, M=2.3, SD=3.7 | 1.12 (1.02 to 1.22) | 0.01 |
| 18 to 20 May 2020 (wave 17) | N=1716, M=2.4, SD=3.0 | 1.14 (1.05 to 1.23) | 0.001 |
| 26 to 27 May 2020 (wave 18) | N=1721, M=2.6, SD=3.4 | 1.25 (1.15 to 1.35) | <0.001 |
| 1 to 3 June 2020 (wave 19) | N=1703, M=2.7, SD=3.2 | 1.28 (1.19 to 1.38) | <0.001 |
| 8 to 10 June 2020 (wave 20) | N=1708, M=3.0, SD=3.9 | 1.41 (1.30 to 1.52) | <0.001 |
| 15 to 17 June 2020 (wave 21) | N=1757, M=3.2, SD=3.9 | 1.52 (1.40 to 1.64) | <0.001 |
| 22 to 24 June 2020 (wave 22) | N=1725, M=3.4, SD=4.2 | 1.62 (1.50 to 1.75) | <0.001 |
| 29 June to 1 July 2020 (wave 23) | N=1726, M=3.4, SD=4.5 | 1.62 (1.50 to 1.76) | <0.001 |
| 6 to 8 July 2020 (wave 24) | N=1712, M=3.5, SD=4.3 | 1.69 (1.56 to 1.82) | <0.001 |
| 20 to 22 July 2020 (wave 25) | N=1717, M=3.9, SD=4.5 | 1.85 (1.71 to 2.00) | <0.001 |
| 3 to 5 August 2020 (wave 26) | N=1723, M=4.2, SD=5.3 | 1.97 (1.82 to 2.14) | <0.001 |
| 1 to 2 September 2020 (wave 27) | N=1745, M=4.2, SD=4.5 | 1.97 (1.83 to 2.13) | <0.001 |
| 14 to 16 September 2020 (wave 28) | N=1719, M=4.2, SD=4.3 | 1.96 (1.82 to 2.11) | <0.001 |
| 28 to 30 September 2020 (wave 29) | N=1702, M=3.9, SD=4.2 | 1.83 (1.69 to 1.97) | <0.001 |
| 12 to 14 October 2020 (wave 30) | N=1780, M=4.0, SD=4.4 | 1.88 (1.74 to 2.02) | <0.001 |
| 26 to 28 October 2020 (wave 31) | N=1728, M=3.7, SD=4.0 | 1.74 (1.61 to 1.87) | <0.001 |
| 9 to 11 November 2020 (wave 32) | N=1713, M=3.4, SD=4.5 | 1.57 (1.44 to 1.70) | <0.001 |
| 16 to 18 November 2020 (wave 33) | N=1779, M=3.4, SD=4.6 | 1.56 (1.43 to 1.70) | <0.001 |
| 23 to 25 November 2020 (wave 34) | N=1731, M=3.4, SD=4.6 | 1.58 (1.45 to 1.72) | <0.001 |
| 30 November to 2 December 2020 (wave 35) | N=1779, M=3.6, SD=4.8 | 1.68 (1.54 to 1.82) | <0.001 |
| 7 to 9 December 2020 (wave 36) | N=1779, M=3.5, SD=4.0 | 1.65 (1.53 to 1.78) | <0.001 |
| 14 to 16 December 2020 (wave 37) | N=1694, M=3.4, SD=4.2 | 1.60 (1.48 to 1.74) | <0.001 |
| 21 to 23 December 2020 (wave 38) | N=1701, M=3.5, SD=4.3 | 1.63 (1.51 to 1.77) | <0.001 |
| 28 to 30 December 2020 (wave 39) | N=1736, M=3.4, SD=4.3 | 1.59 (1.47 to 1.73) | <0.001 |
| 4 to 6 January 2021 (wave 40) | N=1738, M=3.0, SD=3.8 | 1.39 (1.28 to 1.51) | <0.001 |
| 11 to 13 January 2021 (wave 41) | N=1708, M=2.5, SD=3.2 | 1.15 (1.06 to 1.25) | 0.001 |
| 25 to 27 January 2021 (wave 42) | N=1712, M=2.8, SD=4.4 | 1.29 (1.18 to 1.41) | <0.001 |
| 8 to 10 February 2021 (wave 43) | N=1696, M=2.8, SD=3.6 | 1.28 (1.18 to 1.40) | <0.001 |
| 22 to 24 February 2021 (wave 44) | N=1660, M=3.6, SD=5.3 | 1.65 (1.50 to 1.80) | <0.001 |
| 8 to 10 March 2021 (wave 45) | N=1713, M=3.2, SD=4.3 | 1.49 (1.37 to 1.62) | <0.001 |
| 22 to 24 March 2021 (wave 46) | N=1731, M=3.6, SD=5.0 | 1.68 (1.54 to 1.83) | <0.001 |
| 5 to 7 April 2021 (wave 47) | N=1666, M=4.0, SD=4.6 | 1.84 (1.70 to 1.98) | <0.001 |
| 19 to 21 April 2021 (wave 48) | N=1662, M=4.2, SD=4.8 | 1.95 (1.80 to 2.11) | <0.001 |
| 4 to 5 May 2021 (wave 49) | N=1636, M=4.2, SD=4.5 | 1.94 (1.80 to 2.10) | <0.001 |
| 17 to 19 May 2021 (wave 50) | N=1659, M=4.2, SD=4.4 | 1.97 (1.83 to 2.12) | <0.001 |

Table 4. Associations between total outings for shopping, and to see people from another household and survey wave, between June 2021 and January 2022.

| Survey wave | Number of responses, mean number of outings, SD [total n=29,607] | Incidence rate ratio (95% CI) | p-value |
| --- | --- | --- | --- |
| Overall | - | χ^2^(16)=87.7 | <0.001 |
| 1 to 2 June 2021 (wave 51) | N=1697, M=4.9, SD=4.4 | 0.91 (0.85 to 0.97) | 0.004 |
| 14 to 16 June 2021 (wave 52) | N=1670, M=4.8, SD=4.5 | 0.88 (0.82 to 0.94) | <0.001 |
| 28 to 29 June 2021 (wave 53) | N=1704, M=4.8, SD=4.4 | 0.88 (0.82 to 0.94) | <0.001 |
| 26 to 27 July 2021 (wave 54) | N=1732, M=4.9, SD=4.5 | 0.91 (0.85 to 0.97) | 0.004 |
| 9 to 10 August 2021 (wave 55) | N=1706, M=5.1, SD=4.8 | 0.94 (0.88 to 1.01) | 0.08 |
| 23 to 24 August 2021 (wave 56) | N=1719, M=5.2, SD=4.8 | 0.96 (0.90 to 1.03) | 0.22 |
| 6 to 7 September 2021 (wave 57) | N=1723, M=4.9, SD=4.6 | 0.90 (0.84 to 0.96) | 0.003 |
| 20 to 22 September 2021 (wave 58) | N=1632, M=5.3, SD=6.1 | 1.00 (0.93 to 1.08) | 0.97 |
| 4 to 6 October 2021 (wave 59) | N=1685, M=5.4, SD=6.4 | 1.00 (0.92 to 1.08) | 0.96 |
| 18 to 20 October 2021 (wave 60) | N=1590, M=5.2, SD=5.9 | 0.96 (0.89 to 1.04) | 0.34 |
| 1 to 4 November 2021 (wave 61) | N=1833, M=5.3, SD=6.2 | 0.99 (0.92 to 1.06) | 0.70 |
| 15 to 17 November 2021 (wave 62) | N=1864, M=5.9, SD=7.4 | 1.09 (1.01 to 1.17) | 0.04 |
| 29 November to 1 December 2021 (wave 63) | N=1743, M=5.1, SD=6.1 | 0.95 (0.89 to 1.03) | 0.21 |
| 6 to 8 December 2021 (wave 63.5) | N=1679, M=5.5, SD=6.2 | 1.02 (0.95 to 1.10) | 0.60 |
| 13 to 16 December 2021 (wave 64) | N=1841, M=5.3, SD=6.0 | 0.97 (0.90 to 1.05) | 0.48 |
| 4 to 6 January 2022 (wave 65) | N=1876, M=4.8, SD=5.5 | 0.88 (0.82 to 0.95) | 0.001 |
| 17 to 20 January 2022 (wave 66) | N=1913, M=5.4, SD=6.5 | Reference | Reference |

Table 5. Associations between total outings for shopping, to see people from another household, and to go to a restaurant/café/pub and survey wave, between July 2020 and May 2021.

| Survey wave | Number of responses, mean number of outings, SD [total n=46,319] | Incidence rate ratio (95% CI) | p-value |
| --- | --- | --- | --- |
| Overall | - | χ^2^(26)=937.6 | <0.001 |
| 6 to 8 July 2020 (wave 24) | N=1712, M=3.8, SD=4.8 | 0.81 (0.75 to 0.87) | <0.001 |
| 20 to 22 July 2020 (wave 25) | N=1717, M=4.5, SD=5.3 | 0.95 (0.89 to 1.02) | 0.19 |
| 3 to 5 August 2020 (wave 26) | N=1723, M=4.9, SD=6.2 | 1.03 (0.95 to 1.11) | 0.46 |
| 1 to 2 September 2020 (wave 27) | N=1745, M=5.2, SD=5.3 | 1.09 (1.02 to 1.17) | 0.01 |
| 14 to 16 September 2020 (wave 28) | N=1719, M=5.0, SD=5.1 | 1.05 (0.98 to 1.12) | 0.21 |
| 28 to 30 September 2020 (wave 29) | N=1702, M=4.6, SD=5.3 | 0.98 (0.91 to 1.05) | 0.59 |
| 12 to 14 October 2020 (wave 30) | N=1780, M=4.7, SD=5.2 | 0.99 (0.93 to 1.07) | 0.86 |
| 26 to 28 October 2020 (wave 31) | N=1728, M=4.3, SD=4.8 | 0.92 (0.86 to 0.99) | 0.02 |
| 9 to 11 November 2020 (wave 32) | N=1713, M=3.7, SD=5.1 | 0.78 (0.72 to 0.85) | <0.001 |
| 16 to 18 November 2020 (wave 33) | N=1779, M=3.6, SD=5.6 | 0.76 (0.70 to 0.83) | <0.001 |
| 23 to 25 November 2020 (wave 34) | N=1731, M=3.6, SD=5.2 | 0.75 (0.69 to 0.82) | <0.001 |
| 30 November to 2 December 2020 (wave 35) | N=1779, M=3.8, SD=5.8 | 0.81 (0.74 to 0.88) | <0.001 |
| 7 to 9 December 2020 (wave 36) | N=1779, M=3.8, SD=4.5 | 0.81 (0.76 to 0.87) | <0.001 |
| 14 to 16 December 2020 (wave 37) | N=1694, M=3.7, SD=4.5 | 0.80 (0.74 to 0.86) | <0.001 |
| 21 to 23 December 2020 (wave 38) | N=1701, M=3.8, SD=5.1 | 0.81 (0.75 to 0.88) | <0.001 |
| 28 to 30 December 2020 (wave 39) | N=1736, M=3.6, SD=4.8 | 0.77 (0.71 to 0.83) | <0.001 |
| 4 to 6 January 2021 (wave 40) | N=1738, M=3.2, SD=4.4 | 0.67 (0.62 to 0.73) | <0.001 |
| 11 to 13 January 2021 (wave 41) | N=1708, M=2.6, SD=3.7 | 0.56 (0.51 to 0.60) | <0.001 |
| 25 to 27 January 2021 (wave 42) | N=1712, M=2.9, SD=4.9 | 0.62 (0.57 to 0.68) | <0.001 |
| 8 to 10 February 2021 (wave 43) | N=1696, M=2.9, SD=3.9 | 0.61 (0.56 to 0.66) | <0.001 |
| 22 to 24 February 2021 (wave 44) | N=1660, M=3.7, SD=5.6 | 0.78 (0.72 to 0.85) | <0.001 |
| 8 to 10 March 2021 (wave 45) | N=1713, M=3.4, SD=4.9 | 0.72 (0.66 to 0.78) | <0.001 |
| 22 to 24 March 2021 (wave 46) | N=1731, M=3.8, SD=5.4 | 0.80 (0.74 to 0.87) | <0.001 |
| 5 to 7 April 2021 (wave 47) | N=1666, M=4.2, SD=5.0 | 0.87 (0.81 to 0.94) | <0.001 |
| 19 to 21 April 2021 (wave 48) | N=1662, M=4.6, SD=5.3 | 0.98 (0.91 to 1.05) | 0.57 |
| 4 to 5 May 2021 (wave 49) | N=1636, M=4.7, SD=5.1 | 0.99 (0.92 to 1.06) | 0.80 |
| 17 to 19 May 2021 (wave 50) | N=1659, M=4.7, SD=5.0 | Reference | Reference |

Table 6. Associations between total outings for shopping, to see people from another household, and to go to a restaurant/café/pub and survey wave, between June 2021 and January 2022.

| Survey wave | Number of responses, mean number of outings, SD [total n=29,607] | Incidence rate ratio (95% CI) | p-value |
| --- | --- | --- | --- |
| Overall | - | χ^2^(16)=108.6 | <0.001 |
| 1 to 2 June 2021 (wave 51) | N=1697, M=5.7, SD=4.9 | 0.89 (0.84 to 0.95) | 0.001 |
| 14 to 16 June 2021 (wave 52) | N=1670, M=5.6, SD=5.3 | 0.87 (0.82 to 0.93) | <0.001 |
| 28 to 29 June 2021 (wave 53) | N=1704, M=5.7, SD=5.3 | 0.88 (0.82 to 0.94) | <0.001 |
| 26 to 27 July 2021 (wave 54) | N=1732, M=5.9, SD=5.5 | 0.91 (0.85 to 0.97) | 0.007 |
| 9 to 10 August 2021 (wave 55) | N=1706, M=6.1, SD=5.6 | 0.94 (0.88 to 1.01) | 0.07 |
| 23 to 24 August 2021 (wave 56) | N=1719, M=6.2, SD=5.6 | 0.96 (0.90 to 1.03) | 0.24 |
| 6 to 7 September 2021 (wave 57) | N=1723, M=5.8, SD=5.4 | 0.91 (0.85 to 0.97) | 0.005 |
| 20 to 22 September 2021 (wave 58) | N=1632, M=6.4, SD=7.1 | 1.02 (0.95 to 1.10) | 0.57 |
| 4 to 6 October 2021 (wave 59) | N=1685, M=6.5, SD=7.3 | 1.01 (0.94 to 1.09) | 0.70 |
| 18 to 20 October 2021 (wave 60) | N=1590, M=6.3, SD=7.1 | 0.98 (0.91 to 1.06) | 0.61 |
| 1 to 4 November 2021 (wave 61) | N=1833, M=6.4, SD=7.5 | 1.01 (0.94 to 1.08) | 0.83 |
| 15 to 17 November 2021 (wave 62) | N=1864, M=7.1, SD=8.9 | 1.10 (1.02 to 1.19) | 0.01 |
| 29 November to 1 December 2021 (wave 63) | N=1743, M=6.2, SD=7.5 | 0.98 (0.91 to 1.06) | 0.63 |
| 6 to 8 December 2021 (wave 63.5) | N=1679, M=6.6, SD=7.4 | 1.03 (0.96 to 1.11) | 0.36 |
| 13 to 16 December 2021 (wave 64) | N=1841, M=6.4, SD=7.3 | 0.99 (0.92 to 1.07) | 0.84 |
| 4 to 6 January 2022 (wave 65) | N=1876, M=5.8, SD=6.6 | 0.90 (0.84 to 0.96) | 0.003 |
| 17 to 20 January 2022 (wave 66) | N=1913, M=6.4, SD=7.4 | Reference | Reference |

Table 7. Associations between total outings for shopping, to see people from another household, to go to a restaurant/café/pub, and go out to work (in those who reported working) and survey wave, between July 2020 and May 2021.

| Survey wave | Number of responses, mean number of outings, SD [total n=25,097] | Incidence rate ratio (95% CI) | p-value |
| --- | --- | --- | --- |
| Overall | - | χ^2^(26)=572.2 | <0.001 |
| 6 to 8 July 2020 (wave 24) | N=950, M=5.8, SD=6.8 | Reference | Reference |
| 20 to 22 July 2020 (wave 25) | N=960, M=6.6, SD=6.3 | 1.16 (1.07 to 1.27) | 0.001 |
| 3 to 5 August 2020 (wave 26) | N=908, M=7.0, SD=7.4 | 1.23 (1.12 to 1.36) | <0.001 |
| 1 to 2 September 2020 (wave 27) | N=959, M=7.8, SD=7.0 | 1.37 (1.25 to 1.5) | <0.001 |
| 14 to 16 September 2020 (wave 28) | N=940, M=7.5, SD=6.7 | 1.32 (1.20 to 1.44) | <0.001 |
| 28 to 30 September 2020 (wave 29) | N=940, M=7.3, SD=6.7 | 1.28 (1.17 to 1.40) | <0.001 |
| 12 to 14 October 2020 (wave 30) | N=918, M=7.6, SD=6.6 | 1.32 (1.21 to 1.44) | <0.001 |
| 26 to 28 October 2020 (wave 31) | N=868, M=7.0, SD=6.1 | 1.22 (1.12 to 1.34) | <0.001 |
| 9 to 11 November 2020 (wave 32) | N=928, M=6.5, SD=7.6 | 1.14 (1.03 to 1.26) | 0.01 |
| 16 to 18 November 2020 (wave 33) | N=966, M=6.2, SD=7.9 | 1.08 (0.97 to 1.21) | 0.14 |
| 23 to 25 November 2020 (wave 34) | N=936, M=5.9, SD=6.2 | 1.03 (0.94 to 1.14) | 0.50 |
| 30 November to 2 December 2020 (wave 35) | N=967, M=6.3, SD=7.7 | 1.12 (1.01 to 1.24) | 0.03 |
| 7 to 9 December 2020 (wave 36) | N=977, M=6.4, SD=6.2 | 1.12 (1.02 to 1.22) | 0.02 |
| 14 to 16 December 2020 (wave 37) | N=914, M=6.3, SD=5.5 | 1.11 (1.02 to 1.21) | 0.02 |
| 21 to 23 December 2020 (wave 38) | N=907, M=6.2, SD=6.5 | 1.09 (0.99 to 1.20) | 0.10 |
| 28 to 30 December 2020 (wave 39) | N=990, M=5.5, SD=6.5 | 0.96 (0.87 to 1.06) | 0.46 |
| 4 to 6 January 2021 (wave 40) | N=911, M=4.9, SD=6.1 | 0.86 (0.78 to 0.96) | 0.005 |
| 11 to 13 January 2021 (wave 41) | N=950, M=4.5, SD=5.0 | 0.79 (0.71 to 0.87) | <0.001 |
| 25 to 27 January 2021 (wave 42) | N=945, M=4.8, SD=5.5 | 0.84 (0.76 to 0.93) | 0.001 |
| 8 to 10 February 2021 (wave 43) | N=912, M=5.2, SD=5.6 | 0.90 (0.82 to 1.00) | 0.04 |
| 22 to 24 February 2021 (wave 44) | N=904, M=5.8, SD=7.2 | 1.02 (0.92 to 1.13) | 0.73 |
| 8 to 10 March 2021 (wave 45) | N=908, M=5.8, SD=7.0 | 1.02 (0.92 to 1.13) | 0.74 |
| 22 to 24 March 2021 (wave 46) | N=879, M=6.4, SD=6.7 | 1.10 (1.00 to 1.22) | 0.05 |
| 5 to 7 April 2021 (wave 47) | N=915, M=6.7, SD=6.6 | 1.15 (1.05 to 1.27) | 0.003 |
| 19 to 21 April 2021 (wave 48) | N=928, M=7.3, SD=7.1 | 1.28 (1.17 to 1.41) | <0.001 |
| 4 to 5 May 2021 (wave 49) | N=939, M=7.5, SD=6.1 | 1.31 (1.20 to 1.42) | <0.001 |
| 17 to 19 May 2021 (wave 50) | N=878, M=7.6, SD=6.6 | 1.33 (1.22 to 1.46) | <0.001 |

Table 8. Associations between total outings for shopping, to see people from another household, to go to a restaurant/café/pub, and go out to work (in those who reported working) and survey wave, between June 2021 and January 2022.

| Survey wave | Number of responses, mean number of outings, SD [total n=16,087] | Incidence rate ratio (95% CI) | p-value |
| --- | --- | --- | --- |
| Overall | - | χ^2^(16)=135.3 | <0.001 |
| 1 to 2 June 2021 (wave 51) | N=955, M=8.3, SD=5.7 | Reference | Reference |
| 14 to 16 June 2021 (wave 52) | N=869, M=8.7, SD=6.3 | 1.04 (0.98 to 1.11) | 0.18 |
| 28 to 29 June 2021 (wave 53) | N=985, M=8.4, SD=6.0 | 1.01 (0.95 to 1.07) | 0.76 |
| 26 to 27 July 2021 (wave 54) | N=954, M=8.8, SD=6.4 | 1.05 (0.99 to 1.12) | 0.10 |
| 9 to 10 August 2021 (wave 55) | N=922, M=8.8, SD=6.2 | 1.06 (0.99 to 1.13) | 0.08 |
| 23 to 24 August 2021 (wave 56) | N=942, M=8.9, SD=6.1 | 1.07 (1.01 to 1.14) | 0.02 |
| 6 to 7 September 2021 (wave 57) | N=961, M=8.3, SD=5.8 | 1.00 (0.94 to 1.07) | 0.92 |
| 20 to 22 September 2021 (wave 58) | N=849, M=9.7, SD=9.1 | 1.20 (1.11 to 1.29) | <0.001 |
| 4 to 6 October 2021 (wave 59) | N=888, M=9.7, SD=8.6 | 1.17 (1.09 to 1.26) | <0.001 |
| 18 to 20 October 2021 (wave 60) | N=802, M=9.6, SD=8.0 | 1.16 (1.08 to 1.24) | <0.001 |
| 1 to 4 November 2021 (wave 61) | N=959, M=9.8, SD=9.4 | 1.20 (1.11 to 1.29) | <0.001 |
| 15 to 17 November 2021 (wave 62) | N=1031, M=10.4, SD=10.5 | 1.25 (1.16 to 1.35) | <0.001 |
| 29 November to 1 December 2021 (wave 63) | N=943, M=9.6, SD=9.4 | 1.16 (1.08 to 1.25) | <0.001 |
| 6 to 8 December 2021 (wave 63.5) | N=949, M=10.0, SD=8.9 | 1.21 (1.13 to 1.30) | <0.001 |
| 13 to 16 December 2021 (wave 64) | N=1005, M=9.4, SD=8.3 | 1.14 (1.06 to 1.22) | <0.001 |
| 4 to 6 January 2022 (wave 65) | N=1008, M=8.2, SD=8.2 | 0.98 (0.91 to 1.05) | 0.52 |
| 17 to 20 January 2022 (wave 66) | N=1065, M=9.8, SD=9.0 | 1.19 (1.11 to 1.27) | <0.001 |

Table 9. Associations between total outings and national lockdown.

| Total outings for shopping and to see people from another household | | | |
| --- | --- | --- | --- |
| Lockdown | Number of responses, mean number of outings, SD [total n=20,733] | Incidence rate ratio (95% CI) | p-value |
| Overall | - | χ^2^(2)=574.4 | <0.001 |
| First lockdown (30 March to 6 May 2020, waves 10 to 15) | N=10,376, M=2.0, SD=3.0 | Reference | Reference |
| Second lockdown (16 November to 2 December 2020, waves 33 to 35) | N=5289, M=3.4, SD=4.7 | 1.67 (1.60 to 1.75) | <0.001 |
| Third lockdown (25 January to February 2021, waves 42 to 44) | N=5068, M=3.0, SD=4.5 | 1.47 (1.40 to 1.55) | <0.001 |
| Total outings for shopping, to see people from another household, and to go to a restaurant/café/pub | | | |
| Lockdown | Number of responses, mean number of outings, SD [total n=10,357] | Incidence rate ratio (95% CI) | p-value |
| Second lockdown (16 November to 2 December 2020, waves 33 to 35) | N=5289, M=3.7, SD=5.5 | Reference | Reference |
| Third lockdown (25 January to February 2021, waves 42 to 44) | N=5068, M=3.2, SD=4.9 | 0.87 (0.82 to 0.92) | <0.001 |
| Total outings for shopping, to see people from another household, to go to a restaurant/café/pub, and go out to work (in those who reported working) | | | |
| Lockdown | Number of responses, mean number of outings, SD [total n=5,630] | Incidence rate ratio (95% CI) | p-value |
| Second lockdown (16 November to 2 December 2020, waves 33 to 35) | N=2869, M=6.1, SD=7.3 | Reference | Reference |
| Third lockdown (25 January to February 2021, waves 42 to 44) | N=2761, M=5.3, SD=6.1 | 0.86 (0.81 to 0.91) | <0.001 |

Table 10. Associations between frequent wearing of a face covering and survey wave, between April 2020 and May 2021.

| Survey wave | Infrequent hand washing [total n=17,041], n (%) | Frequent hand washing [n=35,196], n (%) | Odds ratio (95% CI) | p-value |
| --- | --- | --- | --- | --- |
| Overall | - | - | χ^2^(36)=8118.2 | <0.001 |
| 27 to 29 April 2020 (wave 14) | 1075 (85.0) | 189 (15.0) | Reference | Reference |
| 4 to 6 May 2020 (wave 15) | 1084 (85.8) | 179 (14.2) | 0.98 (0.79 to 1.22) | 0.88 |
| 11 to 13 May 2020 (wave 16) | 1096 (84.8) | 197 (15.2) | 1.07 (0.87 to 1.32) | 0.52 |
| 18 to 20 May 2020 (wave 17) | 810 (61.3) | 511 (38.7) | 3.74 (3.11 to 4.50) | <0.001 |
| 26 to 27 May 2020 (wave 18) | 835 (62.4) | 503 (37.6) | 3.58 (2.97 to 4.30) | <0.001 |
| 1 to 3 June 2020 (wave 19) | 840 (62.7) | 500 (37.3) | 3.48 (2.90 to 4.19) | <0.001 |
| 8 to 10 June 2020 (wave 20) | 790 (59.4) | 539 (40.6) | 4.03 (3.35 to 4.84) | <0.001 |
| 15 to 17 June 2020 (wave 21) | 795 (57.9) | 577 (42.1) | 4.30 (3.58 to 5.16) | <0.001 |
| 22 to 24 June 2020 (wave 22) | 764 (56.1) | 599 (43.9) | 4.67 (3.89 to 5.60) | <0.001 |
| 29 June to 1 July 2020 (wave 23) | 754 (54.9) | 619 (45.1) | 4.90 (4.09 to 5.88) | <0.001 |
| 6 to 8 July 2020 (wave 24) | 710 (51.4) | 670 (48.6) | 5.59 (4.66 to 6.71) | <0.001 |
| 20 to 22 July 2020 (wave 25) | 610 (42.7) | 820 (57.3) | 8.01 (6.68 to 9.61) | <0.001 |
| 3 to 5 August 2020 (wave 26) | 335 (23.4) | 1095 (76.6) | 19.42 (16.00 to 23.57) | <0.001 |
| 1 to 2 September 2020 (wave 27) | 307 (20.8) | 1171 (79.2) | 22.90 (18.82 to 27.86) | <0.001 |
| 14 to 16 September 2020 (wave 28) | 291 (19.5) | 1202 (80.5) | 24.48 (20.08 to 29.85) | <0.001 |
| 28 to 30 September 2020 (wave 29) | 281 (18.9) | 1207 (81.1) | 25.46 (20.87 to 31.06) | <0.001 |
| 12 to 14 October 2020 (wave 30) | 252 (16.1) | 1313 (83.9) | 30.96 (25.28 to 37.91) | <0.001 |
| 26 to 28 October 2020 (wave 31) | 258 (17.5) | 1220 (82.5) | 27.93 (22.84 to 34.15) | <0.001 |
| 9 to 11 November 2020 (wave 32) | 266 (19.0) | 1134 (81.0) | 25.57 (20.89 to 31.29) | <0.001 |
| 16 to 18 November 2020 (wave 33) | 290 (19.8) | 1177 (80.2) | 24.46 (20.08 to 29.79) | <0.001 |
| 23 to 25 November 2020 (wave 34) | 276 (18.9) | 1182 (81.1) | 25.55 (20.95 to 31.16) | <0.001 |
| 30 November to 2 December 2020 (wave 35) | 270 (17.6) | 1262 (82.4) | 27.75 (22.75 to 33.86) | <0.001 |
| 7 to 9 December 2020 (wave 36) | 277 (18.3) | 1237 (81.7) | 26.52 (21.73 to 32.37) | <0.001 |
| 14 to 16 December 2020 (wave 37) | 237 (16.6) | 1194 (83.4) | 30.27 (24.65 to 37.17) | <0.001 |
| 21 to 23 December 2020 (wave 38) | 250 (17.4) | 1185 (82.6) | 27.94 (22.82 to 34.21) | <0.001 |
| 28 to 30 December 2020 (wave 39) | 258 (18.1) | 1166 (81.9) | 26.79 (21.90 to 32.76) | <0.001 |
| 4 to 6 January 2021 (wave 40) | 199 (14.0) | 1219 (86.0) | 36.51 (29.55 to 45.11) | <0.001 |
| 11 to 13 January 2021 (wave 41) | 255 (18.6) | 1116 (81.4) | 25.95 (21.17 to 31.80) | <0.001 |
| 25 to 27 January 2021 (wave 42) | 262 (19.5) | 1083 (80.5) | 24.68 (20.17 to 30.20) | <0.001 |
| 8 to 10 February 2021 (wave 43) | 245 (17.9) | 1127 (82.1) | 27.39 (22.34 to 33.58) | <0.001 |
| 22 to 24 February 2021 (wave 44) | 236 (17.1) | 1146 (82.9) | 28.73 (23.39 to 35.28) | <0.001 |
| 8 to 10 March 2021 (wave 45) | 285 (19.7) | 1164 (80.3) | 24.41 (20.01 to 29.77) | <0.001 |
| 22 to 24 March 2021 (wave 46) | 290 (19.7) | 1179 (80.3) | 24.10 (19.79 to 29.35) | <0.001 |
| 5 to 7 April 2021 (wave 47) | 326 (22.5) | 1125 (77.5) | 20.53 (16.92 to 24.92) | <0.001 |
| 19 to 21 April 2021 (wave 48) | 319 (22.2) | 1117 (77.8) | 20.75 (17.08 to 25.20) | <0.001 |
| 4 to 5 May 2021 (wave 49) | 300 (21.0) | 1126 (79.0) | 22.09 (18.14 to 26.90) | <0.001 |
| 17 to 19 May 2021 (wave 50) | 313 (21.5) | 1146 (78.5) | 21.71 (17.87 to 26.38) | <0.001 |

Table 11. Associations between frequent wearing of a face covering and survey wave, between October 2020 and May 2021.

| Survey wave | Infrequent wearing of face covering [total n=5,412], n (%) | Frequent wearing of face covering [total n=23,305], n (%) | Odds ratio (95% CI) | p-value |
| --- | --- | --- | --- | --- |
| Overall | - | - | χ^2^(19)=82.3 | <0.001 |
| 26 to 28 October 2020 (wave 31) | 258 (17.5) | 1220 (82.5) | Reference | Reference |
| 9 to 11 November 2020 (wave 32) | 266 (19.0) | 1134 (81.0) | 0.95 (0.79 to 1.14) | 0.58 |
| 16 to 18 November 2020 (wave 33) | 290 (19.8) | 1177 (80.2) | 0.91 (0.76 to 1.09) | 0.32 |
| 23 to 25 November 2020 (wave 34) | 276 (18.9) | 1182 (81.1) | 0.94 (0.79 to 1.13) | 0.52 |
| 30 November to 2 December 2020 (wave 35) | 270 (17.6) | 1262 (82.4) | 1.02 (0.85 to 1.22) | 0.83 |
| 7 to 9 December 2020 (wave 36) | 277 (18.3) | 1237 (81.7) | 0.98 (0.82 to 1.16) | 0.78 |
| 14 to 16 December 2020 (wave 37) | 237 (16.6) | 1194 (83.4) | 1.13 (0.94 to 1.36) | 0.21 |
| 21 to 23 December 2020 (wave 38) | 250 (17.4) | 1185 (82.6) | 1.03 (0.86 to 1.23) | 0.77 |
| 28 to 30 December 2020 (wave 39) | 258 (18.1) | 1166 (81.9) | 0.98 (0.82 to 1.18) | 0.86 |
| 4 to 6 January 2021 (wave 40) | 199 (14.0) | 1219 (86.0) | 1.33 (1.10 to 1.61) | 0.003 |
| 11 to 13 January 2021 (wave 41) | 255 (18.6) | 1116 (81.4) | 0.95 (0.79 to 1.14) | 0.57 |
| 25 to 27 January 2021 (wave 42) | 262 (19.5) | 1083 (80.5) | 0.92 (0.76 to 1.10) | 0.35 |
| 8 to 10 February 2021 (wave 43) | 245 (17.9) | 1127 (82.1) | 1.00 (0.83 to 1.20) | 0.99 |
| 22 to 24 February 2021 (wave 44) | 236 (17.1) | 1146 (82.9) | 1.04 (0.86 to 1.25) | 0.67 |
| 8 to 10 March 2021 (wave 45) | 285 (19.7) | 1164 (80.3) | 0.90 (0.75 to 1.07) | 0.22 |
| 22 to 24 March 2021 (wave 46) | 290 (19.7) | 1179 (80.3) | 0.88 (0.73 to 1.04) | 0.14 |
| 5 to 7 April 2021 (wave 47) | 326 (22.5) | 1125 (77.5) | 0.75 (0.63 to 0.89) | 0.001 |
| 19 to 21 April 2021 (wave 48) | 319 (22.2) | 1117 (77.8) | 0.76 (0.64 to 0.90) | 0.002 |
| 4 to 5 May 2021 (wave 49) | 300 (21.0) | 1126 (79.0) | 0.83 (0.69 to 0.99) | 0.04 |
| 17 to 19 May 2021 (wave 50) | 313 (21.5) | 1146 (78.5) | 0.79 (0.67 to 0.95) | 0.009 |

This analysis was run using an exchangeable correlation matrix.
